# The Analysis of Transcriptomes and Microorganisms Reveals Differences Between the Intestinal Segments of New Zealand Rabbits

**DOI:** 10.3390/ani16030390

**Published:** 2026-01-26

**Authors:** Die Tang, Shuangshuang Chen, Chuang Tang, Xiangyu Li, Mingzhou Li, Xuewei Li, Kai Zhang, Jideng Ma

**Affiliations:** 1 State Key Laboratory of Swine and Poultry Breeding Industry, College of Animal Science and Technology, Sichuan Agricultural University, Chengdu 611130, China; 18163824725@163.com (D.T.); css05052022@163.com (S.C.); etangchuang@foxmail.com (C.T.); xiaoxiangge2025@163.com (X.L.); mingzhou.li@sicau.edu.cn (M.L.); xuewei.li@sicau.edu.cn (X.L.); 2 Sichuan Academy of Grassland Sciences, Chengdu 611743, China; zkscgrassland@163.com

**Keywords:** New Zealand rabbit, transcriptome, 16S, gut

## Abstract

This study systematically characterized functional compartmentalization along the intestinal tract of New Zealand rabbits by analyzing mucosal tissue and luminal contents from distinct segments, including the duodenum, jejunum, ileum, cecum, and colon, using RNA-seq and 16S rRNA sequencing. Transcriptomic analysis revealed that differentially expressed genes identified between the small and large intestines were mainly enriched in digestion, absorption, and immune functions. Genes associated with the transport of amino acids, sugars, vitamins, and bile salts showed significantly higher expression in the small intestine, whereas genes related to water absorption, short-chain fatty acids (SCFAs), nucleotides, and metal ion transport were preferentially expressed in the large intestine. From an immunological perspective, genes involved in fungal responses were enriched in the small intestine, while bacterial response pathways and pattern recognition receptor (PRR) signaling genes were upregulated in the large intestine. Microbiota analysis demonstrated significantly greater diversity and abundance in the large intestine compared with the small intestine. Specifically, Proteobacteria and Actinobacteria were enriched in the small intestine, whereas Firmicutes, Verrucomicrobia, and Bacteroidetes dominated the large intestine. Correlation analysis further identified significant associations between gut microbiota composition and host genes involved in nutrient digestion and absorption. Together, these findings provide transcriptome-based evidence for regional specialization of nutrient transport, immune responses, and microbial ecology along the rabbit intestine.

## Simple Summary

We collected five different intestinal segments from the intestinal tract of New Zealand rabbits, analyzed the key differences in nutrient transport, immune responses, and microbial ecology among different segments of the intestine, and explored the interaction between the transcriptome and microbiota, providing important insights into rabbit intestinal physiology and laying the foundation for understanding host–microbe crosstalk in the New Zealand rabbit model.

## 1. Introduction

The intestine is an organ that integrates immune response [1], digestion and absorption [2,3], resistance to microorganisms [4], and secretion of hormones [5]. Different segments of the intestine exhibit certain specificity in anatomical structure and function. Its anatomical structure is adapted to its function. The duodenum is rich in chyme from the stomach, as well as bile and pancreatic juice secreted by the pancreas and gallbladder. The villi are relatively short, but they have abundant digestive enzymes and absorption surfaces to adapt to rapid digestion and initial absorption. The villi of the jejunum are long and dense, with a large surface area, which is conducive to efficient absorption of nutrients such as glucose, amino acids, and fatty acids. The ileum is the terminal part of the small intestine, mainly responsible for absorbing the remaining nutrients (such as vitamin B12 and bile acids) that are not absorbed by the jejunum. The villi are short and sparse but have abundant lymphoid tissue (such as Peyer’s patches), which plays an important role in immune defense. The surface of the large intestine is covered with dense mucus and lacks villi, so its digestion and absorption ability is weak, making it the main site for microbial fermentation [6,7]. In addition, as the largest immune organ in animals, the intestine also exhibits significant regional specialization in its immune function. The proximal small intestine, as the main site of nutrient absorption, has a high-nutrient-concentration environment and rapid transport characteristics, resulting in a low level of microbial colonization. This region maintains the integrity of the intestinal barrier through the synergistic effects of an acidic microenvironment, an oxygen-rich state, high-concentration IgA secretion, and antimicrobial peptides. As the chyme moves toward the distal small intestine, the decrease in the nutrient concentration gradient drives a significant increase in microbial abundance. The immune features of this region are characterized by the dense distribution of Peyer’s patches (PP), which, together with abundant goblet cells, enhance antigen presentation ability, making the distal small intestine have stronger potential for immune response regulation in microbial–host interactions [8]. In contrast, although the large intestine reduces nutrient transport, it carries the richest microbial community. Through the physical defense mechanism of forming a double-layer dense mucus barrier, it effectively blocks pathogen invasion while allowing symbiotic bacteria to colonize, reflecting the host’s evolutionary adaptation strategy to high-density bacterial environments [9,10].

The regionalization of intestinal function can be influenced by different factors [11], such as gene expression, food, and microbiota. The gut microbiota colonizes from birth to adulthood and tends to stabilize in adulthood [6,12]. The interaction between microorganisms and the gut can affect both the host gut and the microorganisms themselves. For example, *Clostridium butyricum*, as a core member of intestinal symbiotic bacteria, produces high concentrations of butyric acid (up to 20–40 mM in the cecum) by fermenting dietary fiber, directly driving intestinal morphogenesis and functional optimization [13]. The polysaccharide A produced by *Bacteroides fragilis* stimulates tolerant plasma cell–like DCs through Toll-like receptor 2 (TLR2), thereby driving TReg cells that produce IL-10, which may be another mechanism for enhancing colon TReg cell production [14,15]. Some anaerobic *Clostridium* species (including some species in the human intestine) preferentially induce the production of regulatory T cells (TReg) in the colon by promoting an environment rich in transforming growth factor-β (TGF-β) [16,17]. The genomes of certain *Bacteroidetes* bacteria are rich in genes related to carbohydrate degradation, encoding glycoside hydrolases and polysaccharide lyases that contribute to the breakdown of complex carbohydrates [18]. Studies on sterile animals have also shown that gut microbiota can regulate gene expression in the host, such as phase I enzymes, phase II enzymes, transporters, and transcription factors [19,20]. The co-inoculation of *Bacillus subtilis* and *Bacteroides fragilis* into sterile rabbits can promote the proliferation of B cells and the secretion of immunoglobulins, improving the immune function of rabbits [21]. In addition, in germ-free mice, due to the lack of normal stimulation from gut microbiota, the regulation of intestinal wall nerves and muscles is imbalanced, and the cecum often exhibits significant dilation [22]. Slezak et al. [23] also found that the small intestine length of germ-free mice was significantly shorter than that of normal mice. Meanwhile, the colonization of microorganisms is also related to the host itself, and there are species differences in the composition and function of gut microbiota [24].

The New Zealand rabbit is a commonly used model animal in intestinal research. This study describes the intestinal transcriptome and microbial composition of New Zealand rabbits, providing reference data for intestinal research and for the rational use of New Zealand rabbits as experimental models.

## 2. Materials and Methods

### 2.1. Sample Collection

Three healthy 1-year-old male New Zealand White rabbits were obtained from the Sichuan Grassland Science Research Institute and raised under identical feeding and management conditions. All animals were deeply anesthetized prior to euthanasia, which was performed painlessly in strict accordance with institutional and national guidelines for animal welfare and was approved by the local ethics committee. To avoid interference caused by the movement of intestinal contents during dissection, each intestinal segment was ligated with cotton thread before excision to prevent mixing of contents between adjacent segments. During dissection, intestinal mucosal tissues and the corresponding luminal contents from each segment were rapidly and carefully collected. All tissue and content samples were immediately snap-frozen in liquid nitrogen and subsequently stored at −80 °C until further analysis. In total, 45 intestinal mucosal tissue samples were collected for transcriptome (RNA-seq) analysis, including 8 duodenum, 9 jejunum, 10 ileum, 9 cecum, and 9 colon samples. In addition, 34 intestinal content samples were collected for 16S rRNA gene sequencing, comprising 5 duodenum, 4 jejunum, 5 ileum, 9 cecum, and 11 colon samples. Although multiple intestinal segments were sampled, all samples were derived from only three individual rabbits. Therefore, individual animals were considered the true biological replicates.

### 2.2. H&E Staining

Place the paraffin-embedded tissue slices in a 65 °C oven and bake for 2 h to firmly adhere the tissue to the glass slides and complete the pretreatment. Subsequently, dewaxing was carried out by immersing the slices in three containers of xylene (I, II, and III) for 10 min each, followed by transfer to three containers of anhydrous ethanol (I, II, and III) for 10 min each. Afterwards, the slices were immersed sequentially in gradient ethanol solutions of 95%, 85%, and 75% for 5 min each to gradually hydrate the tissue. Finally, the slices were rinsed with tap water for 2 min. During the staining stage, the slices were first placed in hematoxylin staining solution for approximately 30 s (the time can be adjusted according to staining intensity) and rinsed with water for 2 min to complete nuclear staining. Subsequently, the slices were treated with hydrochloric acid–alcohol differentiation solution for 30 s and rinsed with water to complete the blueing process. Afterwards, the slices were placed in eosin staining solution for 2 min (the time can also be adjusted according to staining effect) and then rinsed with water to remove excess stain. Dehydration was performed by rapidly immersing the slices in 75%, 85%, and 95% ethanol for 5 s each, followed by soaking in anhydrous ethanol for 2 min. The slices were then soaked in xylene for 2 min for clearing. During mounting, neutral gum was added onto the tissue and covered with a coverslip. The slides were placed in a fume hood and allowed to stand for 1–2 h to completely evaporate the xylene. Finally, the sealed slides were placed in a digital slide scanner for whole-slide digital scanning.

### 2.3. Total RNA Extraction, Library Preparation and Sequencing

Total RNA was isolated from tissue samples using the HiPure Total RNA Mini Kit (Magen, Guangzhou, China). The integrity and quality of RNA samples were assessed using a NanoDrop 2000 (Thermo Fisher Scientific, Wilmington, DE, USA) and a Bioanalyzer 2100 system (Agilent Technologies, Palo Alto, CA, USA). RNA with an absorbance ratio of 1.8–2.0 at 260/280 nm and a RIN value >7.0 was selected for further study. Library construction was performed by Novogene (Beijing, China), and high-throughput sequencing was conducted on the DNBSEQ-T7 platform. Paired-end sequencing (2 × 150 bp, PE150) was performed, and reads were filtered by removing those containing adapters, polyA or polyG tails, more than 5% unknown nucleotides (N), or more than 20% low-quality bases (Q-value ≤ 20). The quality metrics of sequencing reads are summarized in Supplementary Table S1.

### 2.4. Quantification

Clean data were mapped to the rabbit reference genome *Oryctolagus cuniculus* (OryCun2.0) using STAR (v.2.6.0c). Transcript abundance for protein-coding genes (PCGs) was quantified as transcripts per million (TPM) using Kallisto (v.0.44.0) with default parameters. Expressed PCGs were defined as those with TPM > 1 in at least three samples, taking into account the expression characteristics of different transcripts (lncRNAs with TPM > 0.1 in at least three samples).

### 2.5. Differentially Expressed Genes Analysis and Functional Enrichments

Differential gene expression analysis was performed between pairs of intestinal segments using the edgeR software package (version 4.4.2), and genes with |fold change| > 2 and FDR < 0.05 were considered differentially expressed genes (DEGs). Functional enrichment analysis of DEGs, including Gene Ontology (GO) and Kyoto Encyclopedia of Genes and Genomes (KEGG) analyses, was conducted using the Metascape portal (http://metascape.org, access date: 1 October 2025).

### 2.6. Total DNA Extraction from Microbiome

DNA extraction was conducted using the QIAamp DNA Stool Kit (Qiagen, Hilden, Germany) following the manufacturer’s instructions with slight modifications. Briefly, 100 mg of intestinal contents was weighed and lysed by incubation for 5 min at 95 °C. DNA was eluted with 100 μL of Buffer AE after incubation for 10 min at room temperature. DNA quality was assessed by agarose gel electrophoresis, and DNA concentration was determined using a NanoDrop ND-1000 spectrophotometer (NanoDrop Technologies, Wilmington, DE, USA).

### 2.7. 16S rRNA Gene Amplification and Sequencing

The V3–V4 hypervariable region of the bacterial 16S rRNA gene was amplified by PCR using the universal primers 341F (5′-CCTACGGGNGGCWGCAG-3′) and 805R (5′-GACTACHVGGGTATCTAATCC-3′). The sequencing library was prepared using the TruSeq Nano DNA LT Library Prep Kit (Illumina, Inc., San Diego, CA, USA). First, the amplified products were subjected to end repair using the End Repair Mix 2 from the kit to remove overhanging bases at the 5′ end of the DNA sequence and to add phosphate groups to the 3′ end, filling in any gaps at the 3′ end of the DNA sequence. A base (A) was then added to the 3′ end of the DNA sequence to prevent self-ligation of the DNA fragments and to ensure that the target sequence could be ligated to the sequencing adapters. An indexing adapter containing a library-specific tag was added to the 5′ end of the sequence, allowing the DNA molecule to be anchored to the flow cell. Using BECKMAN AMPure XP Beads (Beckman Coulter, Brea, CA, USA), magnetic beads were utilized to remove unligated fragments and purify the library after adapter addition. The DNA fragments with ligated adapters were then amplified via PCR to enrich the sequencing library templates, and the enriched library product was purified again using BECKMAN AMPure XP Beads. Finally, a 2% agarose gel electrophoresis was performed for the final round of selection and purification.

### 2.8. Methods for 16S rRNA Analysis

For the paired-end data obtained from sequencing, the samples were split based on barcode information, and the adapters and barcode sequences were removed. Subsequently, data concatenation and filtering were carried out, and length filtering and denoising were performed by calling DADA2 through qiime dada2 denoise-paired. Amplicon Sequence Variants (ASVs, features) and ASV (feature) abundance tables were obtained, and singleton ASVs (i.e., ASVs with a total sequence count of only 1 in the entire dataset, default operation) were removed. Alpha diversity and beta diversity analyses were performed based on the obtained ASV (feature) sequences and ASV (feature) abundance tables. According to the ASV (feature) sequence file, species were annotated using the SILVA database with the NT-16S database, and the abundance of each species in each sample was statistically analyzed based on the ASV (feature) abundance table.

### 2.9. Correlation Analysis Between Gut Microbiota and Differentially Expressed Genes

To investigate the potential functional interactions between DEGs in the gut and the gut microbiota, the abundance of microbiota in each intestinal segment was considered as the phenotypic trait, and DEGs from the large and small intestines were extracted and subjected to Spearman rank correlation analysis. Spearman rank correlations, corresponding *p*-values, and significant gene–gene associations (FDR-adjusted *p* < 0.05) were calculated using the corr.test() function in the R package (version 4.4.1) psych.

## 3. Results

### 3.1. Staining Results of Small Intestine Slices

**Figure 1:**
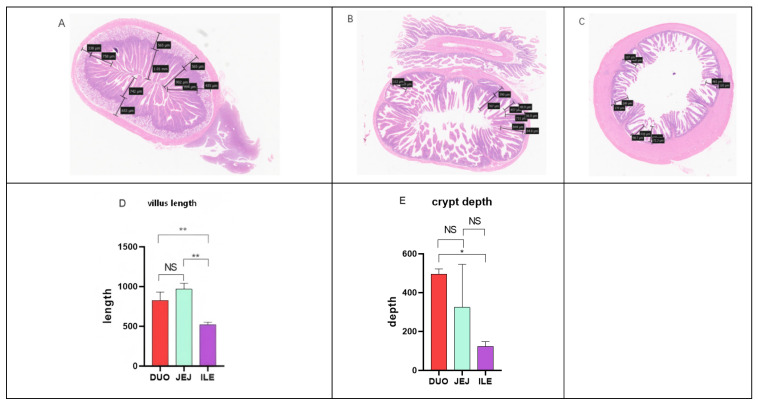
H&E staining and statistical analysis of villus length and crypt depth in various segments of the small intestine (* *p* < 0.05, ** *p* < 0.01, NS: not significant). (**A**) Duodenal (**B**) Jejunal (**C**) Ileum (**D**) Villus length (**E**) Crypt depth.

By analyzing the H&E staining results of rabbit small intestine slices, the overall length of rabbit small intestine villi was observed to follow the order jejunum > duodenum > ileum, and the depth of crypts followed the order duodenum > jejunum > ileum. The average length of villi in the jejunum was 966.3 μm (Figure 1B), in the duodenum was 825.6 μm (Figure 1A), and in the ileum was 519.03 μm (Figure 1C). These structural differences reflect the division of labor and cooperation among different parts of the small intestine in terms of digestion and absorption functions.

### 3.2. Comparison of Transcriptional Profiles of Different Intestinal Segments in New Zealand Rabbits

**Figure 2:**
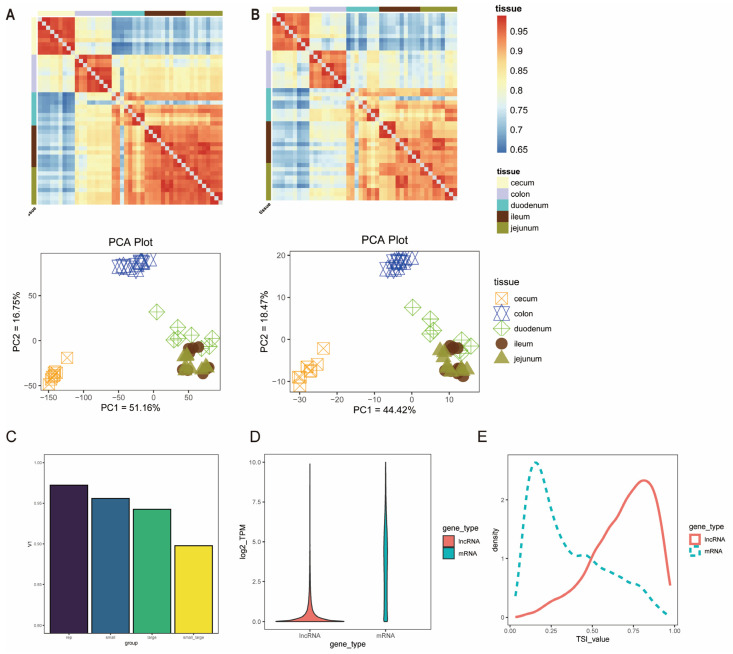
Transcriptome comparison of different intestinal segments. (**A**) Correlation analysis of mRNAs and principal component analysis (PCA). (**B**) Correlation analysis of lncRNAs and principal component analysis (PCA). (**C**) Mean Pearson correlation coefficients within different groups; (**D**) Expression level of PCGs and lncRNAs, TPM value transformed by log2 (TPM + 1); (**E**) Distribution curves of TSI values of PCGs and lncRNAs.

Based on the screened mRNA data, principal component analysis (PCA) was performed. The results showed that PC1 (51.16%) separated the large intestine and small intestine samples significantly, while PC2 (16.75%) mainly reflected region-specific differentiation within the large intestine (Figure 2A). Correlation analysis based on Spearman’s rank correlation coefficient showed that the correlation coefficient between segments of the small intestine reached 0.96, while the correlation coefficient within the large intestine remained at 0.94. The correlation coefficient between the small intestine and the large intestine was 0.90 (Figure 2C), indicating that the large intestine may have higher functional area specialization than the small intestine. Compared with PCGs, the overall transcriptional abundance of lncRNAs was lower (median TPM value: 0.19 vs. 3.59) (Figure 2D). The clustering results obtained from the expression profile of lncRNAs were similar to those of PCGs, and based on the tissue specificity index (TSI), lncRNAs showed higher tissue-specific expression levels than PCGs (Figure 2E).

### 3.3. Functional Differences Between Intestinal Segments

**Figure 3:**
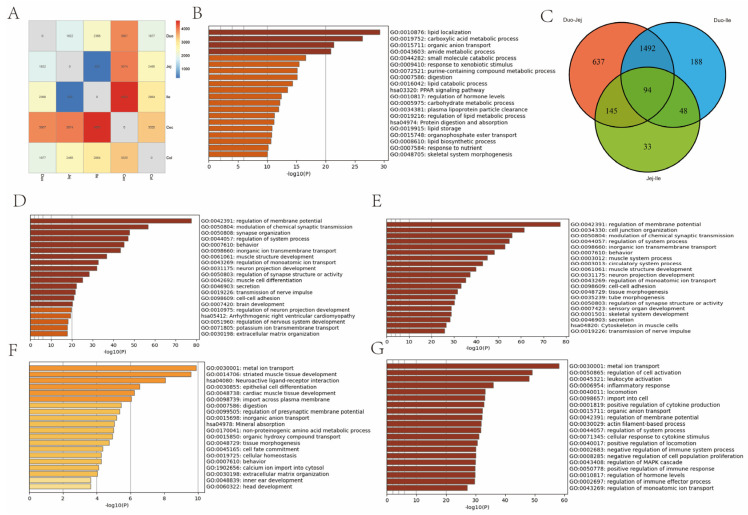
Analysis of Differences in Intestine Size. (**A**) Number of differentially expressed genes. (**B**) Functional enrichment of genes with differences in gut size. (**C**) Wayne diagram showing the number of differentially expressed genes in the small intestine. (**D**–**G**) Functional enrichment of differentially expressed genes within the small and large intestine. (The sequence is Duo-Jej, Duo-Ile, Jej-Ile, Cec-Col).

To investigate the functional differences between different intestinal segments, differentially expressed genes (DEGs) between the large and small intestines were screened using *p* < 0.05 and |fold change| > 2 as criteria. Enrichment analysis of DEGs reflected the functional differences between the large and small intestines, mainly in terms of digestion, absorption, and immune-related functions (Figure 3B).

KEGG and GO enrichment analyses were performed using Metascape. Within the small intestine, there was relatively little difference between the jejunum and ileum, while significant differences were observed between the duodenum and the other small intestine segments. The largest difference was between the duodenum and jejunum (Figure 3A). The differentially expressed genes between the duodenum and jejunum were mainly enriched in secretion (GO:0046903), potassium ion transmembrane transport (GO:0071805), inorganic ion transmembrane transport (GO:0098660), and neuronal development (GO:0031175) (Figure 3D). The differentially expressed genes between the duodenum and ileum were mainly enriched in intercellular adhesion (GO:0098609), secretion (GO:0046903), inorganic ion transmembrane transport (GO:0098660), and muscle structure development (GO:0061061) (Figure 3E). The differentially expressed genes between the jejunum and ileum were mainly enriched in epithelial cell differentiation (GO:0030855), digestion (GO:0007586), metal ion transport (GO:0098660), and mineral absorption (hsa04978) (Figure 3F). These functional differences are consistent with the evolutionary gradient of proximal digestion and distal immunity in the small intestine.

In the large intestine, differentially expressed genes between the cecum and colon were mainly enriched in metal ion transport (GO:0030001), regulation of cell activation (GO:0050865), and organic anion transport (GO:0015711) (Figure 3G).

### 3.4. Expression Pattern of Intestinal Gene Set

#### 3.4.1. Absorption Differences

**Figure 4:**
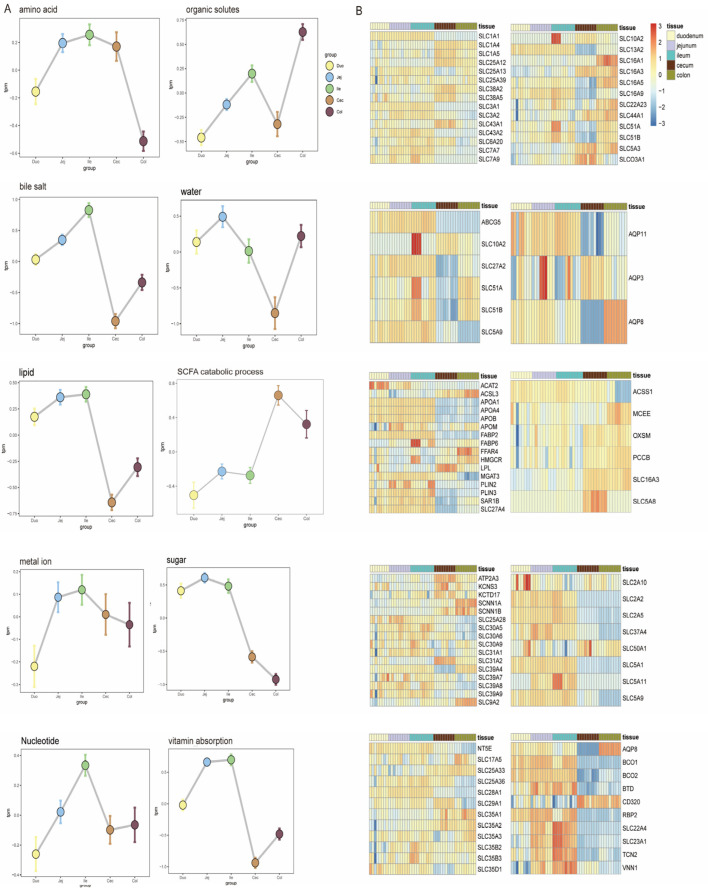
Expression of genes related to nutrient absorption in various intestinal segments. (**A**) Nutrient transport. (**B**) Expression of related genes.

To further investigate the differences between intestinal segments, we downloaded the corresponding functional genes from the QuickGO database and calculated their expression across different intestinal segments, constructing expression profiles of nine common nutrient transport genes in the intestines. Overall, the regional analysis of intestinal absorption function revealed a significant molecular division of labor among different intestinal segments. The small intestine was significantly enriched with multiple categories of nutrient absorption–related gene clusters, including lipid metabolism (fatty acid binding protein family), bile salt transport (ASBT/*SLC10A2*), water-soluble vitamins (SVCT1/*SLC23A1*), and amino acid transport (BAT1/*SLC6A19*). It is worth noting that the monosaccharide transport module presents a refined division of labor, among which fructose permease (*SLC2A5*), sodium-glucose cotransporter (*SLC5A1*), and proton-coupled fructose transporter (*SLC5A11*) were highly expressed in the small intestine, indicating that the small intestine plays a dominant role in the transport of monosaccharides such as glucose and fructose, while the aldose transporter (*SLC50A1*) showed dominant expression in the large intestine, indicating that the large intestine is mainly responsible for the absorption of aldose. The amino acid transport system exhibited chamber differentiation characteristics: neutral and cationic amino acid transporters (such as *SLC7A7* for arginine and lysine) were mainly distributed in the small intestine, while the large intestine specifically enriched L-aspartate transporter *SLC25A12* and Na+-dependent transporters *SLC38A2* and *SLC1A5*. The vitamin transport system showed a bidirectional distribution pattern, with most vitamins, such as the vitamin C receptor (*SLC23A1*)–related genes, highly expressed in the small intestine, while the vitamin B12 receptor (*CD320*) and vitamin A transporter (*AQP8*) were significantly expressed in the large intestine. The core gene *FABP6* for bile acid transport was highly expressed in the ileum, confirming its role as a key site for bile acid absorption (Figure 4B). The expression profile of aquaporins showed that *AQP3* and *AQP11* were mainly localized in the small intestine, while *AQP8* was preferentially expressed in the large intestine. At the same time, the large intestine was enriched with metal ion channels (*KCNS3*, *ATP2A3*, *SCNN1A*) and the nucleotide transporter *SLC35A1*, indicating their important role in maintaining electrolyte balance and microbial metabolite uptake. Short-chain fatty acid–related genes *SLC5A8* and *SLC16A3* were mainly highly expressed in the cecal segment. Despite the homogeneous expression of organic solvent transport genes across various intestinal segments, the overall data still revealed that the small intestine optimizes nutrient absorption through a cascade division of labor, while the large intestine focuses on the transport adaptability for water-salt regulation and microbial interaction products.

#### 3.4.2. Immune Differences

**Figure 5:**
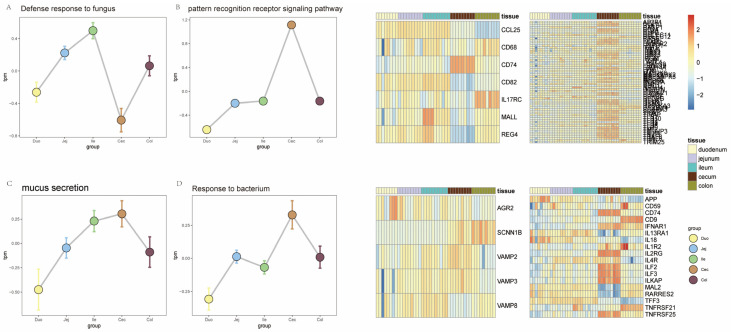
Differences in the immune system across different intestinal segments. (**A**) Expression of genes related to fungal response. (**B**) Expression of genes related to mucus secretion. (**C**) Expression of genes related to pattern recognition receptors. (**D**) Expression of genes related to bacterial response.

The analysis of the intestinal immune defense system showed significant segmental differentiation characteristics (Figure 5). The immune-related gene set covered pattern recognition receptor pathways, bacterial response, fungal defense, and mucus secretion. As an interface for continuous exposure to microorganisms, the gut has evolved a multi-layered defense mechanism: pattern recognition receptors (PRRs) serve as the core components of microbial recognition, including Toll-like receptors (*TLRs*) located on the cell membrane and intracellular NOD-like receptors (*NODs*). Through ligand recognition, downstream signaling cascades are triggered to maintain host–microbe dynamic balance. Expression profiling analysis showed that key components of the pattern recognition receptor signaling pathway (*TLRs*, *NODs*, and transduction protein *MYD88*) were significantly upregulated in the large intestine (Figure 5B), indicating that this region functions as a primary site for immune regulation. In terms of physical barriers, the integrity of the mucus layer is crucial for maintaining the ecological balance of the large intestine microbiota, and its secretion-related gene *SCNN1B* was highly expressed in the large intestine (Figure 5C). Microbial response strategies exhibited regional preference: fungal defense–related genes were mainly enriched in the small intestine, while bacterial response genes showed specific expression in the large intestine (Figure 5A,D), reflecting the differential response mechanisms of different intestinal segments to microbial communities.

### 3.5. Microbiota Differences in Different Intestinal Segments of New Zealand Rabbits

**Figure 6:**
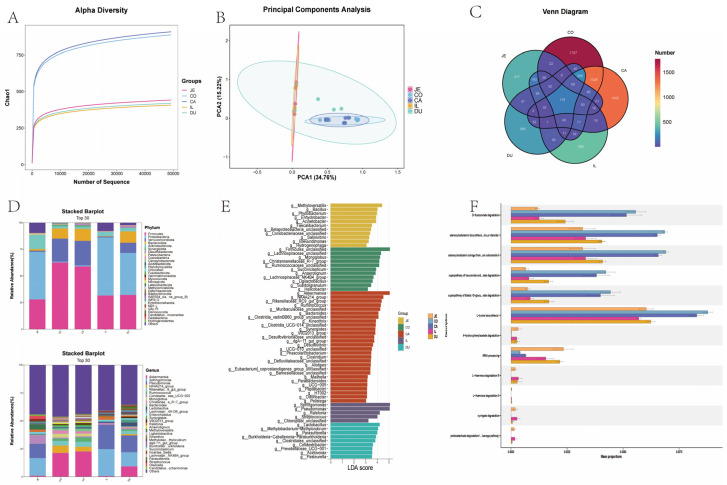
Intestinal microbiota composition. (**A**) Alpha Diversity Index of Intestine (**B**) Clustering of Intestinal Microorganisms (**C**) Venn diagram of microbial abundance in different intestinal segments (**D**) TOP30 Microbial Species in Each Intestinal phylum (**E**) LEFC analysis (**F**) prediction of microbial function in each intestinal segment.

Alpha diversity can effectively reflect the microbiota composition within a sample. The diversity results and Venn diagram showed that both the number and types of microbiota in the large intestine of New Zealand rabbits were much higher than those in the small intestine, with the cecum having the highest number and types of microbiota (Figure 6A,C). Cluster analysis showed that the microbiota in the cecum and colon were similar, whereas the microbiota in the duodenum, jejunum, and ileum were similar (Figure 6B). At the phylum level, Proteobacteria and Actinobacteria were mainly present in the small intestine, while Firmicutes and Bacteroidetes were mainly present in the large intestine, helping to break down indigestible fibers (Figure 6D,E). Subsequently, we predicted gut microbiota function using the MetaCyc database. The predicted functions mainly involved protein degradation and preliminary lignin degradation in the small intestine, such as 4-hydroxyphenylacetic acid degradation, octenoic acid degradation, and proto-naphthalenoic acid degradation, whereas the functions of large intestine microbiota mainly involved cell wall degradation and cellulose/pectin degradation, such as D-fructose degradation and aldehyde glycoside degradation (Figure 6F). These predicted microbial functions also confirmed that the cecum is the main site for microbial fermentation of dietary fiber in rabbits, and the short-chain fatty acids produced by fermentation provide energy for the host.

### 3.6. Correlation Analysis Between Gut Microbiota and Gene Expression

**Figure 7:**
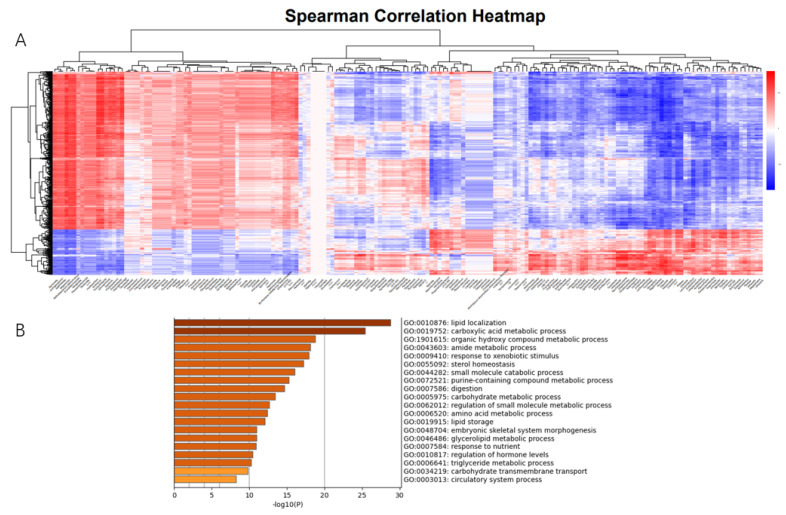
Correlation analysis between gut microbiota and differentially expressed genes. (**A**) Correlation heatmap. (**B**) Enrichment of highly correlated gene functions.

To investigate the correlation between microbiota and gut genes in New Zealand rabbits, we treated the abundance of microbiota in each intestinal segment as a phenotypic trait and selected 660 DEGs with significant differences in abundance between intestinal segments for correlation analysis. We identified 8889 specific and strong microbiota–host associations (Spearman rank correlation ≥ 0.5 or ≤−0.5, FDR-adjusted *p* ≤ 0.01) in the small and large intestine (Figure 7A). Functional enrichment analysis of these highly correlated DEGs showed that they were mainly enriched in digestion and absorption. The enriched pathways included carbohydrate transmembrane transport (GO:0034219), glycerolipid metabolic process (GO:0046486), amino acid metabolic process (GO:0006520), carboxylic acid metabolic process (GO:0019752), organic hydroxyl compound metabolic process (GO:1901615), amide metabolic process (GO:0043603), and purine-containing compound metabolic process (GO:0072521) (Figure 7B).

## 4. Discussion

As the largest immune organ in the body, the intestine integrates digestion, absorption, and immune functions, underscoring its essential role in maintaining host health. Consequently, the intestine has long been a major focus of biological research. Although different intestinal segments participate in digestion, absorption, and immunity, they also exhibit pronounced functional specialization. Several factors contribute to this regional heterogeneity. First, anatomical differences arise during embryonic development, during which the digestive tract establishes an anterior–posterior morphological gradient. Spatial variation in Wnt/β-catenin signaling activity subsequently drives region-specific differentiation of intestinal epithelial cells. Second, transcriptional differences further reinforce this specialization, as single-cell transcriptomic analyses have demonstrated that identical epithelial cell types can perform distinct functions across intestinal segments. In addition, food-derived antigens contribute to regional variation by shaping a dynamic chemical microenvironment during digestion. High concentrations of bile acids in the proximal small intestine activate the farnesoid X receptor (*FXR*), suppress bacterial overgrowth, and induce expression of *SLC10A2*, a bile acid transporter. In contrast, the distal intestine exhibits increased pH and reduced redox potential, promoting a shift in microbial metabolism from glycolysis toward fermentation. Finally, differences in intestinal microbiota further contribute to segment-specific functions, as microbial metabolites can modulate host physiology through metabolic, epigenetic, and immune-related mechanisms. From the perspective of nutrient absorption, the intestine is classically divided into the duodenum, jejunum, ileum, colon, and cecum. However, from an immunological standpoint, finer segmentation is often required, such as distinguishing between the proximal and distal jejunum [25]. Although the cellular composition of each intestinal segment is broadly similar, the functions performed by the same cell types vary markedly across regions [26].

This study employed RNA sequencing and 16S rRNA sequencing to investigate differences among intestinal segments in New Zealand rabbits. RNA-seq analysis revealed pronounced differences in both mRNA and lncRNA expression between the small and large intestines, primarily involving genes associated with digestion, absorption, and immune function. Unlike in other herbivorous animals, the duodenum of New Zealand rabbits is influenced by self-feeding of feces, resulting in gene expression patterns that more closely resemble those of the large intestine, thereby distinguishing it from the jejunum and ileum. In addition, lncRNA expression profiles were found to discriminate more effectively between the small and large intestines than mRNA profiles. This may be attributed to the unique regulatory characteristics of lncRNAs, which do not directly encode proteins but instead influence protein synthesis indirectly by modulating the expression of effector genes [27]. Consequently, lncRNAs display greater spatial heterogeneity across intestinal segments than mRNAs.

To further characterize differences among intestinal segments, we performed differential gene expression analyses across multiple segment combinations and conducted functional enrichment of the identified genes. Distinct differences between the small and large intestines were observed in processes related to digestion and absorption, immune function, and tissue morphogenesis. We also conducted a detailed analysis of the absorption of nine major nutrient categories across intestinal segments. Most nutrients, including sugars, amino acids, and lipids, were predominantly absorbed in the small intestine, whereas water and nucleotides were primarily absorbed in the large intestine. In addition, the small intestine exhibited preferential absorption of specific nutrients. For example, the high expression of sugar transporters such as *SLC2A5*, the sodium–glucose cotransporter *SLC5A1*, and the proton-coupled fructose transporter *SLC5A11* in small intestinal segments suggests a preference for glucose, fructose, and galactose uptake. In contrast, the large intestine appears to preferentially absorb aldoses, as genes involved in aldose transport are predominantly expressed in this region [28,29,30,31]. Neutral amino acids are absorbed in the small intestine via the brush border membrane transporters *SLC6A19* (BAT1) and *SLC43A7* (LAT4), while acidic amino acids are taken up through sodium-dependent transmembrane transport mediated by *SLC1A1* (EAAT3), facilitating glutamate and aspartate absorption. Excitatory amino acids are absorbed through heterodimeric assembly involving *SLC7A7* (y^+^LAT1), enabling leucine/arginine exchange transport. In contrast, the large intestine mediates sodium-coupled amino acid transport through the coordinated activity of *SLC38A2* (SNAT2) and *SLC1A5* (ASCT2), which together facilitate intraluminal uptake of glutamine and alanine, as well as the absorption of aspartate and glutamate [32,33,34]. The terminal ileum achieves efficient reabsorption of approximately 95% of bile acids via *SLC10A2* (ASBT), with transport efficiency further regulated by *FABP6* (IBABP). This protein binds bile acids and activates PPARα/RXR heterodimers, leading to upregulation of *ABCB11* (BSEP) expression, promotion of bile acid efflux from hepatocytes, and completion of the enterohepatic circulation loop [35]. Vitamin transport–related genes are expressed throughout the intestine; however, vitamin absorption occurs predominantly in the small intestine. In contrast, the large intestine primarily regulates intracellular vitamin A transport through *AQP8* aquaporin–mediated membrane permeability.

Faced with a complex intestinal environment, different segments of the intestine have evolved unique immune defense strategies to cope with this microenvironment. The small intestine resists external stimuli through rapid peristalsis and absorption, which reduces contact between microorganisms and food, thereby lowering nutrient availability. A low pH environment (gastric acid secreted by the stomach leads to an acidic environment in the small intestine) inhibits microbial growth. High concentrations of IgA and antimicrobial peptides further support defense; Paneth cells secrete α-defensins HD5/6 (1.2 mg/mL) to target and kill Gram-negative bacteria, while producing approximately 5 g of secretory IgA daily to block microbial adhesion [8]. High IgA concentrations also help maintain the stability of epithelial cell function. Pattern recognition receptor (PRR) activation regulates the expression of tight junction proteins and transporters in the intestinal epithelium. For example, *TLR2*/*TLR4* activation affects the expression of glucose transporters *SGLT1* and *GLUT2*, indirectly regulating carbohydrate absorption. In contrast, the large intestine secretes a large amount of mucus, forming a dense layer that blocks contact between the intestinal surface and microorganisms or food residues. However, an excessively thick mucus layer may partially impair nutrient absorption. Furthermore, defense strategies differ between the small and large intestines of New Zealand rabbits: the small intestine primarily expresses fungal-related genes, whereas the large intestine predominantly expresses bacterial-related genes.

The gut microbiota plays an important role in intestinal function. Colonization begins with the mother’s microbiota during the embryonic stage and continues with external sources, such as food-derived microbiota after birth, until stabilization occurs in adulthood [36]. In addition, the composition of the gut microbiota is influenced by the gut environment; for example, before and after weaning, microbial composition can change due to alterations in the animal’s intestinal environment [37]. Alpha diversity analysis shows that the abundance of microorganisms in the small intestine of New Zealand rabbits is lower than that in the large intestine. Meanwhile, PCA results indicate that the microbial communities in the large intestine are more consistent, suggesting that they are less susceptible to external influences [38]. At the phylum level, Proteobacteria and Actinobacteria are more abundant in the small intestine, whereas Firmicutes, Bacteroidetes, and Verrucomicrobia predominate in the large intestine. *Proteobacteria* is often considered an important indicator for evaluating gut microbiota stability. Although this group lacks the ability to degrade polysaccharides, it is frequently detected in the intestines of animals fed high-calorie, high-fat diets, suggesting it is better adapted to nutrient-rich environments [39]. The role of *Actinobacteria* in nutritional conditions remains controversial. Some studies suggest that a high-fat diet promotes the proliferation of *Actinobacteria*, while other reports indicate that the abundance of this phylum is negatively correlated with body fat percentage [40]. High concentrations of volatile fatty acids (VFAs) fermented by microorganisms in the rabbit cecum, such as acetic acid, propionic acid, and butyric acid, may influence host gene expression through multiple mechanisms. Butyrate esters, for example, have been shown to alter gene transcription in intestinal epithelial cells by regulating histone acetylation, thereby affecting the expression of chemokines and other genes related to immune and barrier functions [41]. We conducted a correlation analysis between microbial abundance and intestinal gene expression. Our analyses suggest that microbial abundance is more closely linked to the expression of digestion- and absorption-related genes. However, these positive correlations need to be further validated using germ-free animals.

Overall, the composition of the large intestine microbiota is highly stable, and its functions are more diverse, enabling it to adapt to complex nutritional environments. In contrast, the microbial community in the small intestine has lower diversity due to stronger nutritional and environmental pressures, relying more on specific microbial populations for metabolism and energy acquisition. These differences indicate that the small and large intestines have complementary microbial functions, with each contributing uniquely to host health. Different dietary habits and nutritional components, such as high-fat or high-fiber diets, can significantly affect the composition of these microbial communities.

A key limitation of this study is its exclusive focus on New Zealand rabbits. Significant differences in gut microbiota exist among different species, which may affect the generalizability of rabbits as model animals. Comparative studies across other animals or humans would provide a broader understanding of intestinal segment differences and microbiota–host interactions. Meanwhile, gut microbiota profiling was performed using 16S rRNA sequencing, which, while informative for community composition, has limited resolution for capturing complex microbial diversity and functional potential. Integrating more comprehensive approaches, such as shotgun metagenomics, could provide higher-resolution insights into microbial taxonomy and function. Finally, the small number of animal samples may affect the credibility of the results. Limited replication reduces statistical power for robust differential gene expression analysis, microbiota comparisons, and host–microbe correlation analyses. Future studies with larger sample sizes are necessary to validate these findings and strengthen the reliability of the conclusions.

## 5. Conclusions

This study presents integrated transcriptomic and microbiota profiles along the intestinal tract of New Zealand rabbits and reveals pronounced segment-specific heterogeneity in gene expression, immune-related features, and microbial composition. The study demonstrates that functional differentiation between intestinal segments is primarily reflected in nutrient digestion and absorption-related genes and immune-associated pathways. Furthermore, correlation analyses identify tight associations between segment-specific microbial communities and host genes involved in nutrient digestion and absorption. Collectively, these findings define key molecular and microbial features underlying intestinal regionalization in rabbits and provide a framework for future mechanistic studies of segment-specific intestinal function and host–microbiota interactions.

## Data Availability

The RNA-seq and 16S rRNA sequencing data are available in the Genome Sequence Archive (GSA; https://ngdc.cncb.ac.cn/gsa, access date: 1 October 2025) under accession numbers PRJCA051134 and PRJCA051134.

## Supplementary Materials

The following supporting information can be downloaded at: https://www.mdpi.com/article/10.3390/ani16030390/s1, Table S1: Reads quality control (QC) table.
